# Clinical complications and anthropometric indicators of chronic kidney disease in a global context: scoping review

**DOI:** 10.1007/s00467-026-07381-9

**Published:** 2026-06-05

**Authors:** Virginia Maria Muniz, Leticia de Paiva Souza Klippel, Karla Laurindo Ferreira Freitas, Karen dos Santos Barbosa, Manoela Cassa Libardi, Olivia Maria de Paula Alves Bezerra, Alexandre Cardoso da Cunha, Alice Pignaton Naseri, Antonio Lima Netto, Haysla Xavier Martins Mendonça, Luciane Bresciani Salaroli

**Affiliations:** 1 Postgraduate Program in Nutrition and Health, Federal University of Espírito Santo, Av. Marechal Campos, 1468 (ou 1440), Campus de Maruípe, Vitória, Espírito Santo 29040-090 Brazil; 2 Postgraduate Program in Public Health, Federal University of Espírito Santo, Vitória, Espírito Santo Brazil; 3 Department of Family Medicine, Mental Health and Public Health, Federal University of Ouro Preto, Ouro Preto, Minas Gerais Brazil; 4 State Health Department of Espírito Santo, Vitória, Espírito Santo Brazil; 5 Postgraduate Program in Social Policy, Federal University of Espírito Santo, Vitória, Espírito Santo Brazil

**Keywords:** Chronic kidney disease, Scoping review, Children, Adolescents, Nutrition

## Abstract

**Background:**

Pediatric chronic kidney disease (CKD) is a serious public health problem and represents a considerable burden on health systems, particularly in low- and middle-income countries (LMICs). Despite well-documented global disparities in CKD management, the extent of these differences across continents remains insufficiently explored, particularly in pediatric populations.

**Objective:**

To explore the body of recent evidence on clinical complications and anthropometric indicators of CKD in childhood and to identify gaps in knowledge that can guide the development of future studies of CKD in childhood, in the global context.

**Data sources:**

Studies were searched in following electronic databases: PubMed/Medline, Embase, Web of Science, and Google Scholar.

**Study eligibility criteria:**

Inclusion criteria consisted of observational quantitative articles (cohort, cross-sectional, case–control studies) in English that evaluated CKD in children. We excluded studies without reported data findings, case studies with fewer than 50 individuals and conducted with the same cohort.

**Participants and interventions:**

Children and adolescents aged 0 to 18 years old with CKD.

**Study appraisal and synthesis methods:**

This research did not aim to evaluate the quality of individual studies, since the main objective of a scoping review is to map the evidence and identify strengths and weaknesses. We conducted a scoping review structured using the methodology developed by Arksey and O'Malley expanded by Levac et al. to map the existing literature. The Preferred Reporting Items for Systematic Reviews and Meta-Analyses for Scoping Reviews (PRISMA-ScR) extension were adopted to increase methodological rigor. Two coders reviewed articles for descriptive information to create summary tables comparing variables of interest.

**Results:**

Fifteen studies met inclusion criteria for this scoping review. Baseline clinical characteristics of individuals were analysed, with 889 from LMICs and 2564 from high-income countries (HICs). Inequality in access to treatment for pediatric patients with CKD between HICs and LMICs was evident.

**Limitations:**

Scoping reviews typically have limitations due to their broad research focus and lack of in-depth analysis of a specific problem; therefore, a meta-analysis is not possible. We limited the included studies to those published in English to avoid translation/interpretation errors. Finally, information biases may be present since the data were mostly extracted from observational studies based on secondary information sources.

**Conclusions and implications of key findings:**

Future studies should:1) Focus on standardizing variable definitions and measurement to reduce heterogeneity in the presentation of results; 2) Explore barriers and facilitators to access to treatment among of children with CKD from LMICs; 3) Promote the development of large multicenter collaborative cohorts of pediatric CKD in LMICs, enabling more representative data, longitudinal follow-up, and evidence-based strategies to improve outcomes.

**Registration number:**

Open Science Framework protocol https://doi.org/10.17605/OSF.IO/Q4HN8

**Graphical abstract:**

A higher resolution version of the Graphical abstract is available as Supplementary information.

**Figure:**
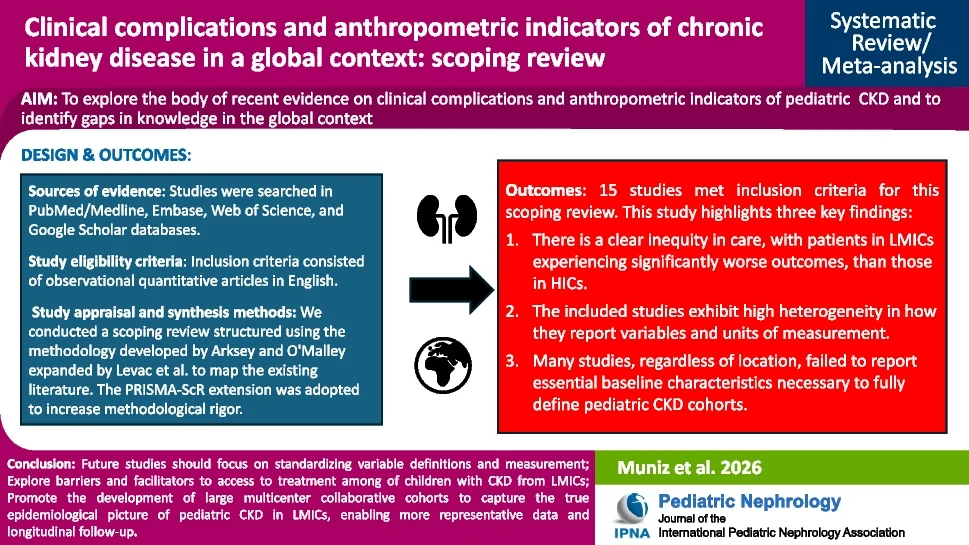

**Supplementary Information:**

The online version contains supplementary material available at https://doi.org/10.1007/s00467-026-07381-9.

## Introduction

Chronic kidney disease (CKD) is diagnosed in adults and children when kidney damage occurs with a normal or increased glomerular filtration rate (GFR) greater than or equal to 90 mL/min/1.73 m^2^, or a reduced GFR less than 90 mL/min/1.73 m^2^, for more than 3 months. Kidney damage is determined by pathological abnormalities or indicators of damage, which include biomarkers in blood or urine biochemical tests, or imaging studies [1]. While global pediatric CKD prevalence is estimated at 15–96 cases per million, epidemiological data remains limited. Despite its lower frequency compared to adult populations, the number of affected children is rising in several regions [2].

Congenital anomalies of the kidney and urinary tract (CAKUT) are the primary cause of pediatric CKD, followed by focal segmental glomerulosclerosis (FSGS), especially in older children. Common complications include multifactorial anemia and hypertension, the latter being a critical risk factor for cardiovascular issues like left ventricular hypertrophy, a leading cause of mortality in this group. Other significant concerns include mineral bone disease associated with chronic kidney disease (MBD-CKD), which affects growth and cardiovascular health, and proteinuria, which serves as both a complication and a biomarker of disease progression. Early diagnosis and management of these comorbidities are essential to improving prognosis and quality of life [3].

The management of MBD-CKD in children is important due to its potential impact on growth and cardiovascular disease. Hyperphosphatemia frequently occurs in moderate-to-severe CKD cases. High dietary calcium intake can induce vascular calcification. Phosphorus retention can occur early in stage 2 CKD due to the consequent reduction in urinary excretion capacity, despite elevated levels of fibroblast growth factor 23 and parathyroid hormone (PTH), which are phosphaturic. Monitoring for reduced vitamin D levels is crucial, as it impairs enteral calcium absorption, leads to the development of secondary hyperparathyroidism, and increases the risk of renal osteodystrophy and pathological fractures. Therefore, supervision by a nutrition specialist focused on kidney disease and rigorous monitoring of anthropometric parameters are pivotal [3].

Compromised nutritional status is common in children with CKD. Previous studies have shown children with malnutrition in 20 to 45% and children with overweight/obesity in 15 to 30% of this population [4]. The recommended anthropometric indices for assessing the nutritional status of children and adolescents with CKD are dry weight, height/length, weight-for-height/length, and head circumference (with the respective z-scores for weight, length, weight-for-length, and head circumference). Anthropometric recording using World Health Organization (WHO) growth charts for all ages or country-specific growth charts for children under 2 years of age is recommended. The frequency of anthropometric monitoring is once every 3 months for children with CKD in stages 2 to 4 and monthly for stage 5 [5].

Pediatric CKD is a serious public health problem and represents a considerable burden on health systems, particularly in low- and middle-income countries (LMICs). This condition tends to progress to total loss of kidney function, requiring kidney replacement therapy (KRT). KRT, in turn, profoundly affects patient’s health and family dynamics, as CKD is often associated with malnutrition, which compromises and delays growth and development, leading to substantial impairments in quality of life and life expectancy. The potential adverse outcomes of CKD can be delayed or prevented by early identification and treatment [6].

The progression of CKD to renal failure necessitates long-term KRT, including kidney transplantation. In addition, serious comorbidities and health complications specific to childhood, such as growth and developmental abnormalities, develop over time. Although the existence of these children-specific problems is evident, there is less evidence for pediatric CKD/renal failure compared with CKD/renal failure in adults, especially in LMICs [7]. A substantial proportion of children with CKD lack access to KRT, highlighting the urgency of developing low-cost treatments and implementing effective prevention strategies at the population level. Despite well-documented global disparities in CKD management, the extent to which these differences reflect variations in risk factors and prevalence across continents remains insufficiently explored, particularly in pediatric populations [8]. In this context, the aim of this scoping review is to explore the body of evidence on clinical complications and anthropometric indicators found in recent observational studies and to identify gaps in knowledge that can guide the development of future studies of CKD in childhood, in the global context.

## Methodological framework

This study conducted a scoping review structured using the methodology developed by Arksey and O'Malley [9] expanded by Levac et al. [10] to map the existing literature. The Preferred Reporting Items for Systematic Reviews and Meta-Analyses for Scoping Reviews (PRISMA-ScR) [11] extension was adopted to increase methodological rigor, with greater transparency and improved reproducibility in reporting the results of this study. The Open Science Framework has registered the review protocol.

### Step 1—Identifying the research question

This study used the PEO framework to construct a research question [12], where "P" is the population; "E" is the exposure; and "O" is the outcome. In this review, "P" refers to children and adolescents aged 0–18 years. The exposure "E" refers to CKD (stages I to V and transplant patients), and "O" refers to clinical and nutritional outcomes (comorbidities such as hypertension, short stature, anemia, bone-mineral disorders, and nutritional status [BMI, BMI z-score, height z-score, weight z-score]). The secondary result was the comparison of the results obtained, according to economic level (LMICs and high-income countries [HICs]) [13] in a global context of pediatric CKD. The research question that guided this scoping review was: What body of evidence is found in the recent literature on the clinical and anthropometric indicators of pediatric patients with CKD in the global context?

### Step 2—Identification of relevant studies and search in electronic databases

Identifying potentially relevant records involved a systematic literature search in three distinct databases: PubMed/Medline, Embase, and Web of Science. Google Scholar was used to retrieve possible additional information from gray literature records. The search was conducted for relevant articles published in the last 10 years (January 2015–October 2025) – this period was chosen because publications about pediatric CKD are scarce and have not yet been fully explored in the last 10 years [2]. Only articles in English were included. The following keywords included in the MeSH terms were used along with Boolean operators: Chronic Kidney Disease "and" children "not" adults. CKD and children not adults. Chronic kidney disease and adolescents not adults, CKD and adolescents not adults. The following limits were used in the advanced search: Type of studies (quantitative, observational); period (last 10 years). References and citations of the full-text records obtained were examined. All records deemed potentially relevant during this research were imported into EndNote 20 software (available at https://endnote.com/); duplicates were systematically eliminated using EndNote's automation tool, and through a manual search to identify and eliminate any duplicates not recognized by the software. Searches were performed in October 2025.

### Step 3—Selection of evidence sources

The selection process involved two distinct stages. Two researchers/coders independently performed the following steps. Any conflicts that arose were resolved by a third author who was not actively involved in the screening process. In the first stage, screening focused on the titles and abstracts of potentially relevant records. Articles related to patients without CKD, studies characterized by a design inconsistent with the eligibility criteria, or in which the population, comparison, or outcome investigated are unclear, were excluded. The full texts of the records identified in the title and abstract screening were retrieved using various approaches, primarily using the dedicated EndNote function. Additional full texts were obtained through internet searches and access to the journals where the studies were published. References and citations in the retrieved full texts were checked to identify additional eligible records.

#### Inclusion and exclusion criteria

Inclusion criteria consisted of observational, quantitative articles (cohort, cross-sectional, case–control studies) in English, with individuals in the pediatric age group (children and adolescents [0 to 18 years old]), with the classification of CKD by Kidney Disease: Improving Overall Outcomes (KDIGO) in I to IV and transplant recipients) [14]. Exclusion criteria were studies involving chronic diseases other than CKD, studies on CKD in adults, quantitative studies such as clinical trials, qualitative studies, opinion studies, book chapters, literature reviews with or without meta-analysis, conference presentation abstracts, case reports, and case studies with fewer than 50 individuals studied, irrelevant studies and studies conducted with the same cohort.

### Step 4—Data extraction process

Two researchers independently performed the data extraction process. Any discrepancies or uncertainties were discussed with a third author until a consensus was reached. Data extracted from the included studies were organized into tables to meet the research objectives, such as: authors, year of publication, country of origin, demographic data (age and sex), clinical aspects such as estimate glomerular filtration rate (eGFR) ml/min per 1.73 m^2^, CKD stage (I–IV) [14], diagnosis of the underlying disease, nutritional indicators (BMI, BMI z-score, height z-score, weight z-score), comorbidities (anemia, hypertension, proteinuria, bone-mineral disorders, short stature), and blood levels of hemoglobin, calcium, phosphorus, albumin, and parathyroid hormone (PTH).

### Step 5: Critical evaluation of individual sources of evidence

Potential biases were identified throughout the mapping process. This research did not aim to evaluate the quality of individual studies, since the main objective of a scoping review is to map the evidence and identify strengths and weaknesses. To achieve this focus, a narrative assessment of the biases and other limitations found in the reviewed literature was conducted. The extracted data were synthesized to highlight similarities and differences. The analysis was conducted to organize and demonstrate the clinical and anthropometric indicators of pediatric CKD across different locations and resource capacities, within a global context, considering the economic status of the country where the study was conducted [13].

## Search results

**Fig. 1:**
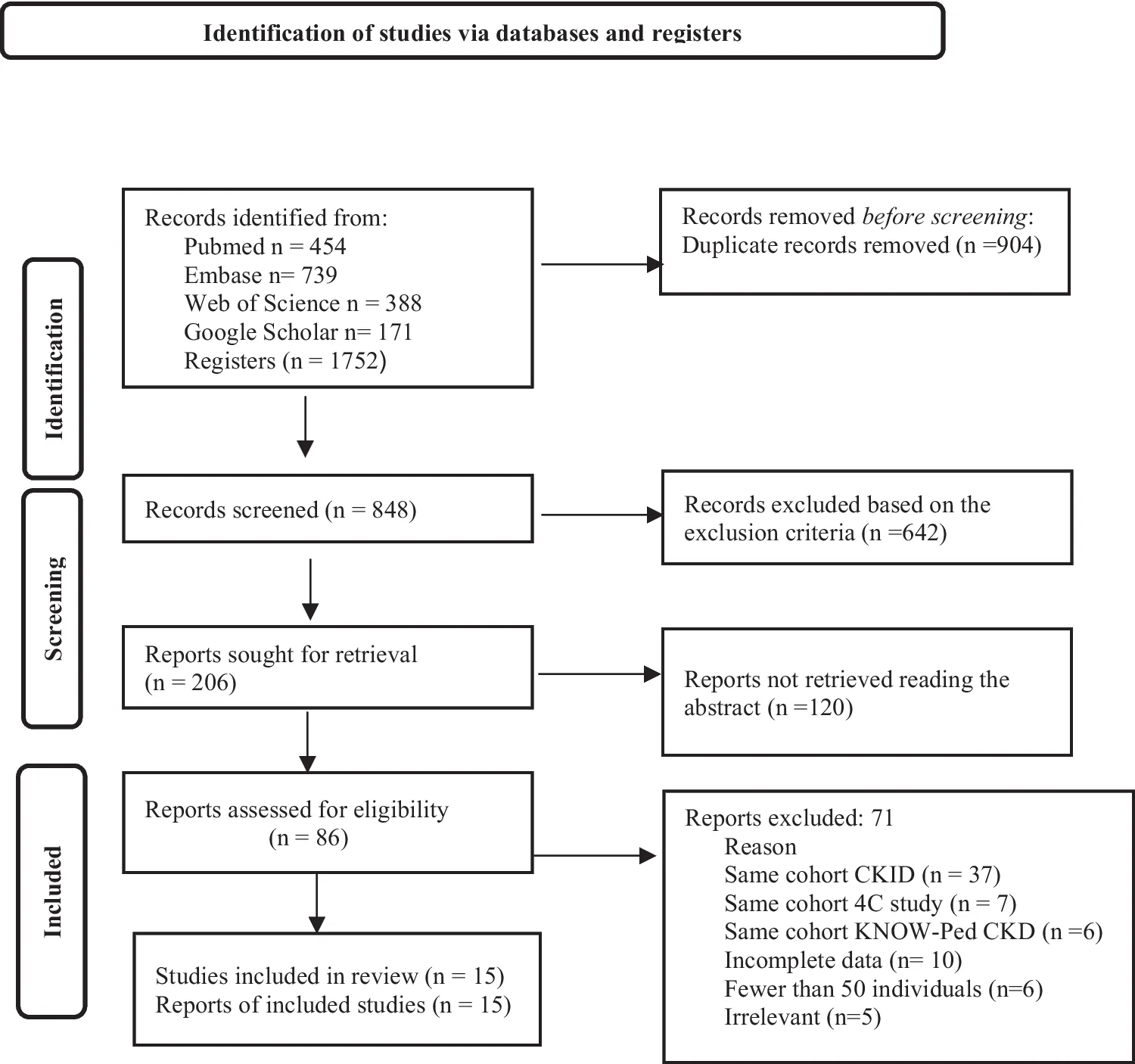
Flowchart for study selection

The PRISMA-ScR flowchart describes the study selection process (Fig. 1)

Fifteen studies met the inclusion criteria for this scoping review [15–29]. It is noted that of the seven studies conducted in HICs, four were cross-sectional and three were prospective multicenter cohorts. The most frequent research problem investigated was the clinical–epidemiological profile, followed by cardiovascular comorbidities and CKD progression. Among the studies conducted in LMICs, all were single-center specialized investigations with a cross-sectional design, and the main research problem was CKD progression, followed by cardiovascular comorbidities and calcium and phosphorus metabolism.

Table 1 presents the most frequently cited baseline characteristics of 2564 pediatric patients with CKD extracted from seven included studies conducted in HICs. Consolidated data indicated that mean ages ranged from 10.1 ± 5.1 to 12.2 ± 3.3 years (yr) [15–17], while medians ranged from 5 (1–9) to 12 (8.5–15) yr [18–20]. In addition, only two studies provided age-stratified data, although they used varying groupings: one study categorized participants into the age ranges of 1 to 4 years (1.1%), 5 to 9 years (33.7%), 10 to 14 years (40.4%), and 15 to 18 years (24.7%) [16]. In contrast, the other study reported distributions for the age ranges < 2 years (9.8%), 2 to 5 years (17.6%), 6 to 11 years (28.2%), and > 12 years (44%) [20]. Males were predominant in all studies [15–21]. Five studies showed a predominance of CKD stages III to V (55% to 100%) [15, 17, 19–21], however, in two studies stage II predominated (73% to 88.3%) [16, 18]. The GFR ml/min per 1.73 m^2^ was verified by seven studies [15–21] with mean variations between 18.3 ± 15.4 ml/min per 1.73 m^2^ and 124.6 ± 52.9 ml/min per 1.73 m^2^ and medians of 53.1 (31.5–78.1) ml/min per 1.73 m^2^ and 83 (62.2–99.6) ml/min per 1.73 m^2^. The methodology for estimating the eGFR varied across the included studies. Two studies [15, 19] used the original 1976 Schwartz formula [30], while two others [17, 21] applied the updated 2009 version [31]. One study [18] used an age-stratified approach, employing the 1987 formula [32] for infants under one year and the 2009 equation for older children. Another study [16] prioritized the 1987 Schwartz formula when height records were available, defaulting to the height-independent eGFR-Pottel calculation [33] otherwise. Finally, one study [20] primarily utilized the creatinine–cystatin C-based CKiD equation [34], substituting the 2009 Schwartz formula for patients lacking cystatin C data. CAKUT was the prevalent underlying diagnosis in four studies (54.2% to 89%) [15, 17, 20, 21]; glomerular disease was prevalent in one study [16]. However, the authors of this latter study observed a possible failure in reporting of cases by the services that treated them, most of which were surgical. Five studies reported rates of non-glomerular diseases among 12.4% to 100% [16–19, 21]. Three investigations reported on anthropometric indicators of BMI z-score [15, 18, 20] and four described the height z-score [15, 19–21]. Both nutritional indexes were within the adequate range for age. Other anthropometric measurements such as BMI mean (± SD) [17], weight mean (± SD) [16, 18], height mean (± SD) [16, 18] were cited in some studies. Two studies reported short stature in their patients, with percentages of 8.7% and 31% [16, 19]. Notably, only one study reported growth hormone therapy, which was administered to 68% of its participants [19]. Hypertension was the most frequent comorbidity reported, with rates ranging from 30.4% to 71% [15, 16, 18–21], followed by anemia with rates between 23.2% and 30.4% [16, 18–20]. Only one study reported average hemoglobin level of 12.7 g/dl [17]. Serum albumin levels were analysed by three studies with means (SD) of 3.8 ± 0.5 g/dl and median (interquartile range [IQR]) of 4.3 (3.9–4.5) gr/dl [19, 21]. Proteinuria rates were verified by three studies [16, 18, 20] and ranged from 34 (19%) to 42 (51.2%). Regarding MBD-CKD parameters, the following biochemical markers were analysed: serum calcium was reported in three studies [15, 17, 21] with means values ranging from 2.2 ± 0.3 to 2.37 ± 0.10 mmol/L. Additionally, one study identified hypocalcemia in 4.5% of the participants [19]. Serum phosphate was reported by three studies, and the mean values ​​ranged from 1.42 ± 0.22 to 1.8 ± 0.6 mmol/L [15, 17, 21]. One study reported hyperphosphatemia in 16% of the patients included [19]. Serum 25-hydroxyvitamin D3 levels were reported in three studies: two found median values ranging from 19.9 (13.6, 37.5) to 26.1 (17.3, 42.7) ng/mL, while the third reported a mean of 62.3 ± 32.3 nmol/L [15, 17, 20]. Four studies reported PTH levels using varying units of measurement with the following medians: 91.8 (45–116) ng/L, 54.2 (29.2–99.3) pg/mL, and 12.8 (7.4–23.6) pmol/L [15, 20, 21] and a mean of 62.2 ± 89.4 pmol/L [17]. To address this heterogeneity, reference ranges are provided for each unit of measurement in the footnotes.

**Table 1:** Baseline characteristics of children with CKD extracted from studies conducted in HICs

Study	Sinha et al., 2015 [15]	Tan et al., 2016 [16]	El Desoky et al., 2016 [17]	Chen et al., 2019 [18]	Brown et al., 2020 [19]	Parker et al., 2022 [20]	Doyon et al., 2025 [21]
Country	United Kingdom	Brunei Darussalam	Arabia	Taiwan	USA/ Canadaª	South Korea	4 C Study^b^
Income	HIC	HIC	HIC	HIC	HIC	HIC	HIC
N/%	83 (100)	89 (100)	119 (100)	308 (100)	858 (100)	437 (100)	670 (100)
Age (yr)^c^	12.1 ± 3.2	11.3 ± 4.12	10.1 ± 5.1	5 (1,9)	12 (8.5,15)	10.92 (5.2,14.6)	12.2 ± 3.3
Male sex, n (%)	51 (61.4)	51 (57.3)	72 (60.5)	169 (55)	542 (63)	297 (67.9)	442 (66)
Stage I–II, n (%)		76 (88.3)		227(73)	306 (36)	196 (47)	
Stage III–V, n (%)	83 (100)	10 (11.6)	119 (100)	51(16.5)	552 (64)	224 (53)	670 (100)
Nonglomerular disease, n (%)		28 (31.4)	61 (51)	308(100)	603 (70)		83 (12.4)
Glomerular disease, n (%)		61 (68.5)	14 (12)		255 (30)	112 (25.6)	59 (8.8)
Unknown/others, n (%)	38 (45.8)	8 (9.0)	24 (20)	12 (3.9)		139 (31.8)	63 (9.4)
CAKUT n (%)	45 (54.2)	20 (22.5)	20 (16.8)	275 (89)		186 (42.6)	465 (69.4)
BMI score z (kg/m^2^)^c^	0.53 ± 1.16			0.17 (–0.51,1.21)			0.11 ± 1.27
BMI^c^			16.8 ± 4.3				
Height z-score^c^	− 0.46 ± 1.28				–0.4 (–1.3, –0.4)	− 0.7 (− 1.82,–0.15)	–1.32 ± 1.33
Height^c^		123.7 ± 27.6		107.15 (82.25,136.4)			
eGFR ml/min per 1.73 m^2 c^	32.3 ± 14.6	124.6 ± 52.9	18.3 ± 15.4	83.07 (62.2,99.6)	55.5 (40.5,69.5)	53.1 (31.5,78.1)	27.0 ± 11.5
Anemia n (%)		19 (23.2)		47 (24)	228 (27)	158 (36.1)	
HB (g/dL) ^c^		12.7(1.65)					
Hypertension n (%)	59 (71)	27 (34.2)		69 (34.5)	460 (54)	115 (30.4)	372 (55.5)
Proteinuria n (%)		42 (51.2)		34(19)		78(17.8)	
Short stature n (%)		23(31)			75 (8.7)^§^		
Serum Ca^c^	2.37 ± 0.10^*^		2.2 ± 0.3^*^		39 (4.5)^£^		2.26 ± 0.19^*^
Serum P^c^	1.42 ± 0.22^*^		1.8 ± 0.6^*^		137 (16)^¥^	347 (79.5)^¥^	1.55 ± 0.37^*^
25(OH)D3	26.1 (17.3,42.7)^**^		62.3 ± 32.3^***^			19.9 (13.6,37.5)^**^	
Serum iPTH	91.8 (45,116)^$^		62.2 ± 89.4^#^			54.2 (29.2,99.3)^&^	12.8 (7.4,23.6)^#^

*HIC* High Income Country, *yr* Year, *CAKUT* Congenital Abnormalities of the Kidney and Urinary Tract, *BMI* Body mass index, *eGFR* estimated glomerular filtration rate, *HB* Hemoglobin, *Ca* Calcium, *P* Phosphate, 25 (OH) D3 25-hydroxyvitamin D3, *PTH* parathyroid hormone

^a^CKiD—54 tertiary care pediatric nephrology programs across North America

^b^4C-study—55 pediatric nephrology units across 12 European countries (Austria, Czech Republic, France, Germany,

Italy, Lithuania, Portugal, Serbia, Switzerland, Turkey, United Kingdom)

^c^median/interquartile range (IQR) or mean ± standard deviation (SD)

^§^individuals using growth hormone

^£^report of hypocalcemia (n/%)

^¥^report of hyperphosphatemia (n/%)

^*^Ca mmol/L (normal range 2.1 to 2.7 mmol/L)

^*^P mmol/L (normal range 1.3 to 2.3 mmol/L

^**^25 OH D3 ng/ml (normal range 24 to 86 ng/L)

^***^ 25 OH D3 nmol (normal range 50 to 125 nmol)

^$^ng/L (normal range 10 to 65 pg/mL)

^&^pg/mL (normal range10 to 65 pg/mL)

^#^pmol/L (normal range1.1 to 6.9 pmol/L)

Table 2 presents the most frequently cited baseline characteristics of 889 pediatric patients with CKD extracted from eight studies included in this paper conducted in LMICs. Consolidated data indicated that mean ages ranged from 4.73 ± 3.7 yr to 10.7 ± 4.8 yr [23, 25, 27, 28], while medians ranged from 9 (5.7–13) yr to 15.28 (11.0–16.6) yr [22, 24, 26, 29]. However, the most recent results from a Brazilian study showed that the median number of years between the onset of the disease and the start of KRT was 1.2 years (0.6–1.9) and for transplantation was 3.3 (1.5–6) yr [24]. Furthermore, only one provided age-stratified data, grouping participants into three categories: 0.3–2 years (36%), > 2–10 years (56%), and > 10–14 years (8%) [29]. Males were predominant in six of the eight studies [22, 24–28]. Regarding clinical characteristics, most individuals investigated were in stages III to V of CKD, with percentages ranging from 72.5% to 100% [22–29]. The eGFR was verified by five studies [22, 23, 25, 27, 28] with average variations between 26.7 ± 10.2 ml/min per 1.73 m^2^ and 47.5 ± 14 ml/min per 1.73 m^2^ and medians of 34.67 (24.3–65.4) ml/min per 1.73 m^2^ and 41 (16–65) ml/min per 1.73 m^2^; in one study all individuals had kidney failure [24]. To calculate eGFR, five studies [22, 23, 26–28] utilized the 2009 Bedside Schwartz equation [31]. The remaining studies relied on older versions: one [25] used the original 1976 formula [30] and another [29] used the 1987 update [32]. Notably, one study [24] focused specifically on pediatric transplant patients. CAKUT was the prevalent baseline diagnosis, varying from 37 to 79% among six studies [22–26, 28, 29]. Regarding anthropometric indicators, two studies reported using the BMI z-score [22, 24] and three the height z-score [22–24]. The mean (SD) or median (IQR) of the BMI z-score was within the normal range for age. One study from India showed that the height z-score (median IQR) had low values for age [22]. Other means (SD) of anthropometric measurements such as BMI [23, 25, 28], weight [24, 25] and height [24, 25] were cited in some studies. Short stature was reported by three studies, with percentages ranging from 27.2% to 78% [22, 26, 27]. No studies from LMICs reported GH use, although one cited high costs as a primary barrier to local availability. Hypertension was the most frequently reported comorbidity, with rates ranging from 38 to 66% [22, 24–29], followed by anemia (37% to 51%) [22, 25–28]. Hemoglobin levels found were 10.3 ± 1.0 g/dl and 11.18 ± 2.0 g/dl [25, 28], however, in one study the median was 11.2 (9.9–12.8) g/dl [22]. Serum albumin levels were analysed by two studies with mean of 4.0 ± 0.60 g/dl [28] and one study found a median of 4.3 (3.6–4.8) g/dl [24]. Proteinuria rates were verified by three studies and varied from 34 to 83% [22, 26, 29]. Reported MBD-CKD markers included serum calcium with mean of 8.2 ± 0.62 to 9.5 ± 0.7 mg/dL [23, 25] and ionized calcium mean of 2.2 ± 0.2 mg/dL; median of 1.27 (1.20,1.34) mg/dL) [24, 28]. Additionally, one study found that 51% of patients presented with hypocalcemia [29]. Three studies assessed phosphate level [23, 25, 28]. One study reported a lower average of 1.75 ± 0.45 mg/dL [28] and one study reported a median of 5 (4.3,5.1) mg/dL [24]. Furthermore, two studies identified hyperphosphatemia in 31% and 47% of their respective patient cohorts [22, 29]. Across five studies, 25-hydroxyvitamin D3 levels varied significantly. Median values ranged from 13.46 ng/ml (8.66,18.54) to 26.5 ng/ml (22.34,30.66) [22, 24], while means spanned from 13.7 ± 2.6 ng/ml to 49.5 ± 23 ng/ml [23, 25, 28]. Notably, one study reported a 79% prevalence of hypovitaminosis D among participants [29]. This table also includes variations in units of measurement in the footnotes.

**Table 2:** Baseline characteristics of children with CKD extracted from studies conducted in LMICs

Study	Kamath et al., 2017 [22]	Coccia et al., 2017 [23]	De Menezes et al., 2019 [24]	Ali et al., 2021 [25]	Amanullah et al., 2022 [26]	van Biljon et al., 2024 [27]	Huarong et al., 2025 [28]	Wannous et al., 2025 [2]
Country	India	Argentina	Brazil	Egypt	Pakistan	South Africa	China	Syria
Income	LMIC	UMIC	UMIC	LMIC	LMIC	UMIC	UMIC	LIC
n (%)	78 (100)	135 (100)	68 (100)	60 (100)	229 (100)	125 (100)	94 (100)	100 (100)
Age (yr)^a^	9 (5.7,13)	10.7 ± 4.8	15.28 (11.0,16.6)	7.05 ± 2.74	10 (5,13)	4.83 ± 2.3	10 (6,12)	4.73 ± 3.7
Male sex, n (%)	58 (74)	72 (43)	42 (62)	32 (53.3)	147 (64.2)	91 (73)	65 (69.1)	48 (48)
Stage I–II, n (%)	21 (27)	34 (25)			63 (27.5)	30 (27)	29 (31)	6 (6)
Stage III–V, n (%)	57 (73)	101 (75)	68 (100)	60 (100)	166 (72.5)	95 (73)	65 (79)	94 (94)
Nonglomerular disease, n (%)	54(69.2)	66 (49)	32 (47)	22 (37)	188 (79)	81 (65)	62 (66)	79 (79)
Glomerular disease, n (%)	12 (15.3)	21 (16)	18 (26.5)	38 (63)	34 (15)	44 (35)	21 (22.3)	21 (21)
Unknown/others, n (%)	25 (25)	48 (35)	18 (26.5)		13 (5.7)		11 (11.7)	
CAKUT, n (%)	37 (47.5)	66 (49)	28 (41)	22 (37)	112 (49)		52 (55.3)	79 (79)
BMI z-score ^a^	− 1.00 (− 2.00,–0.12)		− 0.66 (− 1.49, 0.42)					
BMI^a^		18.6 ± 5.2		14.95 ± 1.3			18.8 ± 20.4	
Height z-score ^a^	− 2.17 (− 3.31, –1.16)	–1.7 ± 1.6	− 1.51 (− 2.30, − 0.68)					
Height ^a^			142.93 ± 26.87	111.5 ± 18.0				
eGFR ml/min per 1.73 m^2 a^	34.67 (24.3, 65.4)	40.5 ± 39		26.7 ± 10.2		47.5 ± 14	41 (16,65)	
Anemia, n (%)	29 (37)			22.5 (37.5)	100 (45)	62 (49.6)		51 (51)
HB (g/dL) ^a^	11.2 (9.9,12.8)			10.3 ± 1.0			11.18 ± 2.0	
Hypertension n (%)	51 (66)		45 (66)	39 (65)	82 (38)	69 (55)	47 (50)	56 (56)
Proteinuria n (%)	73 (94)				39 (58.9)	56 (45)		
Short stature n (%)	51 (65)				170 (78)^+^	34 (27)^§^		
Serum Ca^a^		9.5 ± 0.7^&^	1.27^&$^ (1.20,1.34)	8.2 ± 0.62^&$^			2.2 ± 0.2^&$^	51 (51)^£^
Serum P^a^	25 (32)^¥^	5 ± 1^&^	5 (4.3,5.9)^&^	5.7 ± 1^&^			1.75 ± 0.47^&^	47 (47)^¥^
25(OH)D3^a^	13.46^#^ (8.66,18.54)	27.1 ± 12.9^#^	26.5 (22,34)^#^	13.7 ± 2.6^#^			49.5 ± 23^#^	79 (79)^∞^
Serum PTH	124.11^*^ (60.5,229.9)	118^*^ (66,324)	339^*^ (105,704)	386.5 ± 122^?^			54.6^**^ (27.5,123)	65 (65)^€^

*LMIC* Low-Middle Income Country, *LIC* Low-Income Country, *UMIC* Upper-Middle Income Country, *yr* Year, *CAKUT* Congenital Abnormalities of the Kidney and Urinary Tract, *BMI* Body Mass Index, *eGFR* estimated Glomerular Filtration Rate, *HB* Hemoglobin, *Ca* Calcium, *P* Phosphate, 25 (OH) D3 25-hydroxyvitamin D, *PTH* parathyroid hormone

^a^Median (Interquartile Range IQR) or mean ± Standard Deviation SD

^£^Report of hypocalcemia (n/%)

^¥^Report of hyperphosphatemia (n/%)

^∞^Report of Vitamin D deficiency (n/%)

^€^Report of hyperparathyroidism (n/%)

^+^Height less than the third percentile or standard deviation for height score more negative than –1.88

^§^The authors reported stunted:34 (below 10th percentile); severely stunted: 30 (below 5th percentile)

^*^PTH pg/mL (normal range:10 to 65 pg/mL)

^**^PTH pmol/L (normal range: 1.1 to 6.9 pmol/L)

^&^Ca mg/dL (normal range: 8.8–10.8 mg/dL)

^&^P mg/dL (normal range: 4.0 to 6.5 mg/dL)

^#^25 OH D3 ng/ml (normal range 24 to 86 ng/L)

^$^ionized calcium

^?^without a unit of measurement

The outcomes observed in the included studies from HICs were: the independent predictive factor found for hyperparathyroidism was eGFR at presentation of CKD [17]; elevated systolic blood pressure, proteinuria, and anemia were risk factors associated with CKD progression in patients with non-glomerular disease [18]; low bicarbonate levels were associated with a higher risk of CKD progression [19]; a significant longitudinal increase in carotid intima-media thickness (cIMT SDS) z-score was observed in children with CKD [21]; there was an association between Fibroblast Growth Factor 23 and left ventricular mass in children with non-dialysis stages of CKD [15]; there was a high prevalence of CKD in the pediatric population, and glomerulonephritis was the most frequent causative disease [20]. Finally, a descriptive study demonstrated a high prevalence (736 per million child population [pmcp]) and incidence (91 pmcp) of CKD, and the baseline characteristics found were similar to those of studies conducted in other countries [16]. Furthermore, the outcomes investigated in the studies from LMICs were: in two studies, late presentation of CKD increased treatment costs [22, 26]; among CKD patients, 32% had 25(OH)D deficiency, which reached 51% among those with stage 5 CKD (dialysis). Predictors of deficiency included hypoalbuminemia, advanced CKD, and place of origin [23]; CAKUT were the main cause of CKD and progression was faster in glomerular diseases, and modifiable risk factors, such as hypertension and proteinuria, play an important role in CKD progression, and this demonstrates the importance of early detection and appropriate management of these factors [29]; modifiable risk factors in CKD such as anemia, hypertension, and acidosis were not associated with CKD progression in prepubertal children [27]; regardless of whether ambulatory blood pressure was normal, a reduction in the dipping in blood pressure was correlated with the risk of renal progression in children with CKD [28]; arterial calcification is not frequent in the pediatric age group with kidney failure [24]; bone mineral density biomarkers were significantly associated with hypertrophy and increased left ventricular mass index [25].

## Discussion

This scoping review highlights three key findings from recent observational studies on pediatric CKD. First, there is a clear inequity in care, with patients in LMICs experiencing significantly worse outcomes, particularly regarding MBD-CKD markers, than those in HICs. Second, the included studies exhibit high heterogeneity in how they report variables and units of measurement. Finally, many studies, regardless of location, failed to report essential baseline characteristics necessary to fully define pediatric CKD cohorts.

This study confirmed that the effects of CKD in resource-limited regions are still poorly explored [26]. The number of individuals analysed in research conducted in HICs was triple that of the LMICs. This result may be due to the creation of multicenter cohorts carried out in HIC, such as Chronic Kidney Disease in Children Study (CKiD) in North America, KoreaN cohort study for Outcomes in patients With Pediatric Chronic Kidney Disease (KNOW-Ped CKD) in South Korea, and The Cardiovascular Comorbidity in Children with Chronic Kidney Disease Study (4C Study) in 12 European countries [19–21], while in LMICs all studies came from single-center cross-section. The small number of individuals with childhood CKD found in publications from LMICs, compared to studies in HIC, does not represent a lower actual prevalence, but rather a combination of underdiagnosis, underreporting, and limitations in access to medical care and research infrastructure [35].

Individuals with pediatric CKD constitute a heterogeneous age range, but diagnosis or progression to more advanced stages mainly occur during adolescence [36, 37]. The ages described in this study refer to those at the time of data collection. It is observed that in LMICs the average ages were lower than in HICs. In general, in HICs the age range is broad, but some medians and means reported in studies in three cohorts are around 10 to 14 years [19–21]. Children with congenital diseases tend to progress more slowly and may be diagnosed early, although other glomerular causes may emerge later in childhood or adolescence [36]. In contrast, in LMICs, late-onset CKD is more common, and predominantly associated with acquired rather than congenital causes [38]. Stratifying pediatric CKD by age is essential because its impact on development varies significantly across life stages. Infants and young children face a higher risk of severe complications due to early onset. Additionally, renal function in children under two requires specialized staging with age-specific GFR values, differing from the standards used for adolescents and adults [2].

Most individuals in the eight studies conducted in LMICs were in stages of CKD III to V (72.5 to 100%). In two studies conducted in HICs, most individuals were in stages I and II (73% and 88.3%). This fact reflects the higher values of eGFRs in HICs compared to LMICs. Late diagnosis is a global finding among children with CKD, with higher rates reported in studies in LMICs. Late presentation of CKD mainly results in unfavourable clinical outcomes and greater economic burden for regions with limited health resources [26, 38]. It is noted that the method for calculating eGFR varied among the included studies, with the Schwartz 2009 formula predominating. Measuring eGFR is key to determining the severity of CKD. In pediatric patients, this is especially difficult because eGFR estimation requires more variables than just age and sex. Because children are constantly growing, shifting muscle mass makes creatinine-based estimates less reliable. This often masks the early stages of kidney decline, which typically show no clinical signs [39].

The main cause of CKD in children is CAKUT, accounting for more than 50% of cases [40]. In this present study CAKUT was the most prevalent underlying cause of diagnosis in both HICs and LMICs. The fundamental difference in the occurrence of CAKUT in LMICs compared to HICs lies in the disease burden, age at diagnosis, access to specialized services, and outcomes, particularly progression to kidney failure and mortality. In LMICs, CAKUT were associated with a higher overall disease burden and worse prognosis, due to late diagnosis and limited access to specialized care available in HICs [41].

It has already been demonstrated that children with CKD exhibit significant difficulties in achieving adequate nutritional balance and linear development. Given that a compromised nutritional status can lead to unsatisfactory growth, its assessment is essential in the clinical management of pediatric patients with CKD. The etiology of inadequate linear growth involves hormonal dysregulation of growth, malnutrition, metabolic acidosis, anemia, mineral bone disease, and inflammation [42]. The z-score for height was the most frequently cited and in a study conducted in India this average indicated very low stature in that cohort of patients. There was a lack of reports on the use of growth hormone, especially in HIC; only one cohort included GH in its therapeutic approach, while in LMICs one study cited the high cost of this medication. Furthermore, heterogeneity in presentation of data related to the growth of patients with CKD makes it difficult to conduct systematic reviews. Several initiatives have been established globally to standardize a core set of outcomes and their measurement and selection that can be used in research and activities to improve the quality of studies on childhood CKD [43].

In advanced pediatric CKD, effective management of uremia and related comorbidities, such as anemia, bone and mineral disorders, and cardiovascular diseases is fundamental to delaying the progression to CKD and improving overall quality of life [44]. Hypertension was the most frequently reported comorbidity in the included studies; however, the values found were similar in both LMICs and HICs. Currently, the prevalence of hypertension in childhood in a global context cannot be verified precisely because of the lack of defined international standards [45]. Two large cohorts of pediatric CKD followed in the US and Europe found hypertension rates in children with CKD between 25 and 50% [46]. Hypertension itself is responsible for worsening renal function and causes left ventricular hypertrophy. Cardiovascular diseases are the leading cause of death in children with CKD [3]. Anemia was the second most frequently reported comorbidity in the included studies, and its rates were higher in LMICs. If untreated, anemia increases the risk of hospitalization, left ventricular hypertrophy and overall morbidity in pediatric CKD [3].

The biochemical markers of MBD-CKD were verified in ten of fifteen included studies. It is noted that PTH was above expected values ​​in all reports, as were vitamin D deficiency, hyperphosphatemia, and hypocalcemia, although with significant variation in the units of measurement. The heterogeneity in the presentation of biochemical test results related to calcium, phosphorus, and PTH made it difficult to convert the values as recommended by KDIGO [14]. However, it is noted that PTH values ​​are much higher in studies originating from LMICs. This result may indicate an inequity in the diagnostic approach to pediatric CKD between HICs and LMICs. In many regions, families battling CKD face a heavy financial toll because their essential medications are often left out of standard healthcare coverage [47]. Besides, one group of researchers suggested establishing a core outcome set to be reported in all trials conducted in children with any stage of CKD, and this initiative could also be effective for observational studies [43].

The burden of CKD in children is unknown and underreported because many resource-poor regions and health systems lack renal registries. Furthermore, the nonspecific and late-onset symptoms often lead to CKD being overlooked, particularly in the early stages of the disease. In resource-poor settings, CKD in children is commonly associated with reduced or absent renal function, late hospitalization, and a lack of healthcare facilities providing the necessary and appropriate renal care [48]. From the outcomes reported in the included studies, it was noted that in LMICs the late presentation of CKD, with increased treatment costs, mortality and comorbidities, was frequent, while in HIC the most reported outcomes were related to disease progression and comorbidities.

This scoping review provided a comprehensive overview and mapped the body of recent evidence available, including indexed and gray literature. There are some limitations to our study, which will be pointed out below. First, scoping reviews typically have limitations due to their broad research focus and lack of in-depth analysis of a specific problem, therefore, a meta-analysis is not possible. Second, we limited the included studies to those published in English to avoid translation/interpretation errors. Third, data gaps regarding key variables in the included studies may have introduced bias and constrained the overall synthesis of evidence. Finally, information biases may be present since the data were mostly extracted from observational studies based on secondary information sources.

In conclusion, this review mapped the relevant and current evidence on baseline characteristics of pediatric patients with CKD in a global context. Inequality in healthcare management is noted, according to the country's income level. Future studies should: 1) Focus on standardizing variable definitions and measurement to reduce heterogeneity in the presentation of results. 2) Explore barriers and facilitators to access to treatment among children with CKD from LMICs. 3) Promote the development of large multicenter collaborative cohorts to capture the true epidemiological picture of pediatric CKD in LMICs, enabling more representative data, longitudinal follow-up, and evidence-based strategies to improve outcomes.

## Supplementary Information

Below is the link to the electronic supplementary material.

Supplementary file1Graphical abstract (PPTX 79 KB)

Supplementary file2 (DOCX 85 KB)

## Data Availability

Data sharing not applicable to this article as no datasets were generated or analysed during the current study.
